# Contractile Effects of Glucagon in Mouse Cardiac Preparations

**DOI:** 10.3390/ijms27010126

**Published:** 2025-12-22

**Authors:** Joachim Neumann, Franziska Schmidt, Pauline Braekow, Uwe Kirchhefer, Jan Klimas, Katarina Hadova, Ulrich Gergs

**Affiliations:** 1 Institute for Pharmacology and Toxicology, Medical Faculty, Martin-Luther-University Halle-Wittenberg, Magdeburger Straße 4, D-06097 Halle, Germany; franziska.schmidt.ue@gmail.com (F.S.); pauline.braekow@student.uni-halle.de (P.B.); ulrich.gergs@medizin.uni-halle.de (U.G.); 2 Institute for Pharmacology and Toxicology, Medical Faculty, University Münster, Domagkstraße 12, D-48149 Münster, Germany; kirchhef@uni-muenster.de; 3 Department of Pharmacology and Toxicology, Faculty of Pharmacy, Comenius University in Bratislava, Odbojárov 10, SK-832 32 Bratislava, Slovakia; jan.klimas@uniba.sk (J.K.); hadova@fpharm.uniba.sk (K.H.)

**Keywords:** glucagon, atrium, Langendorff-heart, adult mouse, cardiac preparations

## Abstract

Glucagon is an endogenous peptide that is produced in the pancreas. Via glucagon receptors, glucagon increases the beating rate in cultured rat neonatal cardiomyocytes and also in isolated right atrial preparations from adult rats. Moreover, in living adult mice, injections of glucagon can elevate the heart rate. It is unknown whether these effects of glucagon in living adult mice are mediated via central glucagon receptors or via a direct effect on cardiac glucagon receptors. Thus, we tested the hypothesis that glucagon can exert a direct positive chronotropic effect in the adult mouse heart. We measured the contractile effects of cumulatively increasing concentrations of glucagon (0.1–100 nM) in isolated paced (1 Hz) left atrial preparations, in isolated spontaneously beating right atrial preparations and in isolated spontaneously beating retrogradely perfused whole hearts. We detected in isolated right atrial preparations time- and concentration-dependent positive chronotropic effects of glucagon that were reversed by the glucagon receptor antagonists SC203972 and desglucagon. The positive chronotropic effects of glucagon were also attenuated by 1 µM of ivabradine, an inhibitor of the hyperpolarization-activated cation channels (HCN), but not by 100 nM rolipram, a phosphodiesterase 4 inhibitor, nor by 10 µM of propranolol, a β-adrenoceptor antagonist. Moreover, the positive chronotropic effects of glucagon were also attenuated by stimulation of the A_1_-adenosine receptor or muscarinic receptors. Glucagon decreased the force of contraction in right atrial preparations. In left atrial preparations, glucagon failed to alter the force of contraction. In isolated adult mouse hearts perfused in the Langendorff mode, 10 nM of glucagon increased the beating rate and reduced left ventricular force of contraction. The gene expression of the glucagon receptors was lowest in the left atrium, higher in the ventricle and highest in the right atrium of adult mice. In summary, glucagon exerted a positive chronotropic effect in the mouse heart via glucagon receptors, mediated, at least in part, via HCN channels in the sinus node.

## 1. Introduction

**Figure 1:**
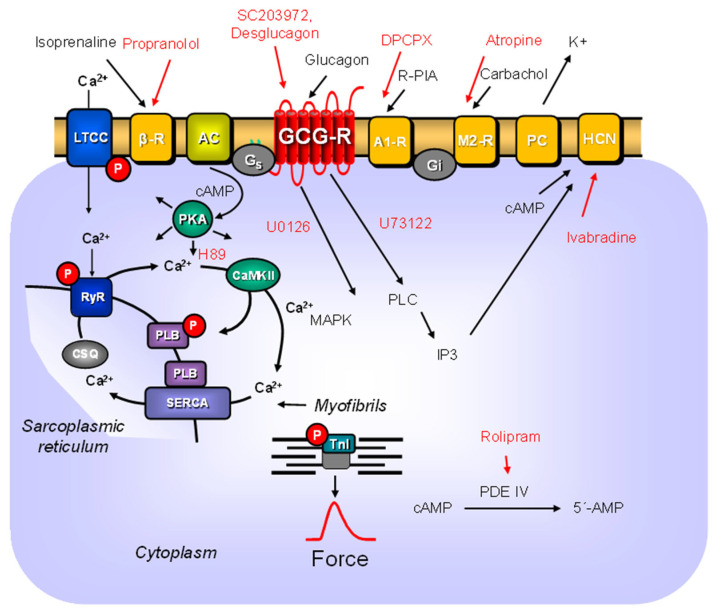
Scheme on glucagon signal transduction.

Glucagon, a peptide comprising 29 amino acids, is produced in α-cells in pancreatic islets of Langerhans (e.g., [1]). Glucagon acts via glucagon receptors (Figure 1, GCGR [2,3,4,5,6]). GCGR raises the activity of adenylyl cyclases in many tissues via stimulatory guanosine-triphosphate-binding proteins (G_s_-proteins) [7]. In principle, glucagon has positive inotropic and positive chronotropic effects in the heart, but these effects show species differences and age differences [8,9,10,11]. Here, we were interested in the cardiac effects of glucagon in adult mice. For instance, others reported that glucagon, in contrast to isoprenaline, failed to stimulate the activity of adenylyl cyclases in adult mouse cardiac membranes [12]. In adult mice, one might distinguish between effects of glucagon on the heart in vivo and ex vivo. Under in vivo conditions, injection of glucagon increased the beating rate in adult wild-type mouse hearts [13]. Injected glucagon failed to augment the beating rate in living adult transgenic mice with constitutive ablation of the GCGR [13]. This suggests that the chronotropic effects of injected glucagon in vivo in the adult mouse are GCGR-mediated [13]. However, in these adult transgenic mice, the GCGR was not only deleted in the heart but also in the brain [13]. Hence, glucagon might have worked in that study via the brain GCGR. Such a central effect of glucagon might have resulted from an enhanced activity of the sympathetic outflow from the brain. Indeed, GCGR is present in the mouse brain, e.g., [14]. Moreover, others have studied the influence of glucagon in isolated Langendorff-perfused hearts from adult wild-type mice [15]. In that study, glucagon did not alter left ventricular pressure, but the authors did not report the beating rate after infusion of glucagon. Hence, a conceivable positive chronotropic effect of glucagon might have been overlooked [15]. Therefore, we tested here the hypothesis that glucagon exerts a direct positive chronotropic and/or inotropic effect in the isolated, spontaneously beating right atrial preparations or isolated hearts from adult wild-type (CD1) mice. We decided to study mice because the genome of mice is currently often manipulated to elevate or reduce the expression of genes of interest as models for the human heart (e.g., [15]). For example, mice with general deletion of the GCGR have been published [16]. Moreover, in recent reports, GCGR antagonists could ameliorate heart failure in adult mice [17,18,19]. Thence, it seems mechanistically important to understand the direct cardiac effects of glucagon and GCGR antagonists in the isolated atrium of adult mice. From a clinical perspective, the cardiac GCGR is gaining interest as a drug target for the treatment of cardiac diseases. This principle is used in the drugs retatrutide, mazdutide, survodutide and cotadutide [20]. These drugs activate the GCGR. Fittingly, central GCGR stimulation can reduce appetite, slow gastric emptying and can increase the energy consumption in the human body. For this reason, stimulation of the GCGR might be helpful to improve the efficacy of new drugs that reduce the body weight and thus increase life expectancy. Hence, a better understanding of the GCGR in the heart and its signal transduction seems to be clinically desirable.

As mentioned, GCGR is thought to act mainly via stimulation of adenylyl cyclase. In the mouse heart, the β-adrenoceptors (using isoprenaline as an agonist) like GCGR act mainly via stimulation of adenylyl cyclases (Figure 1). Now, stimulation of A_1_-adenosine receptors in the presence of isoprenaline leads to a negative chronotropic effect in the adult mouse atrium in part via inhibition of adenylyl cyclases (e.g., [21]). Similarly, stimulation of muscarinic receptors in the presence of isoprenaline leads to a negative chronotropic effect in the mouse atrium, also in part via inhibition of adenylyl cyclases (e.g., [21]). We wanted to know whether a similar interaction between GCGR and muscarinic receptor agonists (using carbachol) or adenosine A_1_-receptor agonists (using R-PIA) in the mammalian atrium exists. Moreover, the positive chronotropic effect of isoprenaline probably occurs via direct stimulation of HCN channels by cAMP (Figure 1, [22]). If glucagon acted via cardiac GCGR, then this effect should be reversed by GCGR antagonists. Here, we decided to study antagonists from two chemical classes: a peptide (desglucagon) and a small organic molecule (SC203972). In addition, we wanted to find out whether glucagon acted via stimulation of the cAMP-dependent protein kinase, via the release of noradrenaline from cardiac stores, via an inhibition of phosphodiesterases, via a stimulation of MAP kinase or via a stimulation of phospholipase C: such pathways exist for GCGR at least in non-cardiac tissues.

Glucagon stimulates the glucagon receptor (GCG-R, blocked by SC203972 and desglucagon) which leads to an increase of adenylyl cyclase (AC) activity through stimulatory GTP-binding proteins (Gs). Isoprenaline activates AC via the β-adrenoceptors (β-R, inhibited by propranolol). AC increases the formation of 3′,5′-cyclic adenosine monophosphate (cAMP). This cAMP stimulates the cAMP-dependent protein kinase (PKA, inhibited by H89). PKA phosphorylates (red P in circles) and thus activates inter alia phospholamban (PLB), the inhibitory subunit of troponin (TnI), the ryanodine receptor (RYR) and the L-type calcium channel (LTCC). The cAMP can directly activate the If-currents carried by hyperpolarization-activated cation channels (HCN channels, blocked by ivabradine). The formed cAMP can be degraded to inactive 5′-AMP by phosphodiesterases (PDEs) in the mouse heart mainly via PDE IV (inhibited by rolipram). Calcium cations (Ca^2+^) are stored by binding to calsequestrin (CSQ) in the sarcoplasmic reticulum and are released via RYR from the sarcoplasmic reticulum. This released Ca^2+^ binds to troponin C on thin myofilaments, and as a result, the force is augmented. In cardiac diastole, Ca^2+^-concentrations fall because Ca^2+^ is pumped into the sarcoplasmic reticulum via a calcium ATPase (SERCA). The activity of SERCA is increased when phospholamban (PLB) is phosphorylated. The activation of GCGR can be functionally antagonized by A_1_-adenosine receptors (A1-R, stimulated by R-PIA and antagonized by DPCPX) or by M_2_-muscarinic receptors (M2-R, stimulated by carbachol and antagonized by atropine). The GCGR may activate phospholipase C (PLC, inhibited U73122), which forms inositoltrisphosphate (IP3) and MAPK (inhibited by U0126). M2-R and A1-R may in part act via opening potassium channels (PCs).

Hence, our main hypotheses were as follows:

1. Glucagon increases the beating rate in the isolated adult mouse heart.
2. This is mediated by cardiac GCGR acting through stimulation of HCN channels.

Parts of this study have been published as abstracts [23,24].

## 2. Results

**Figure 2:**
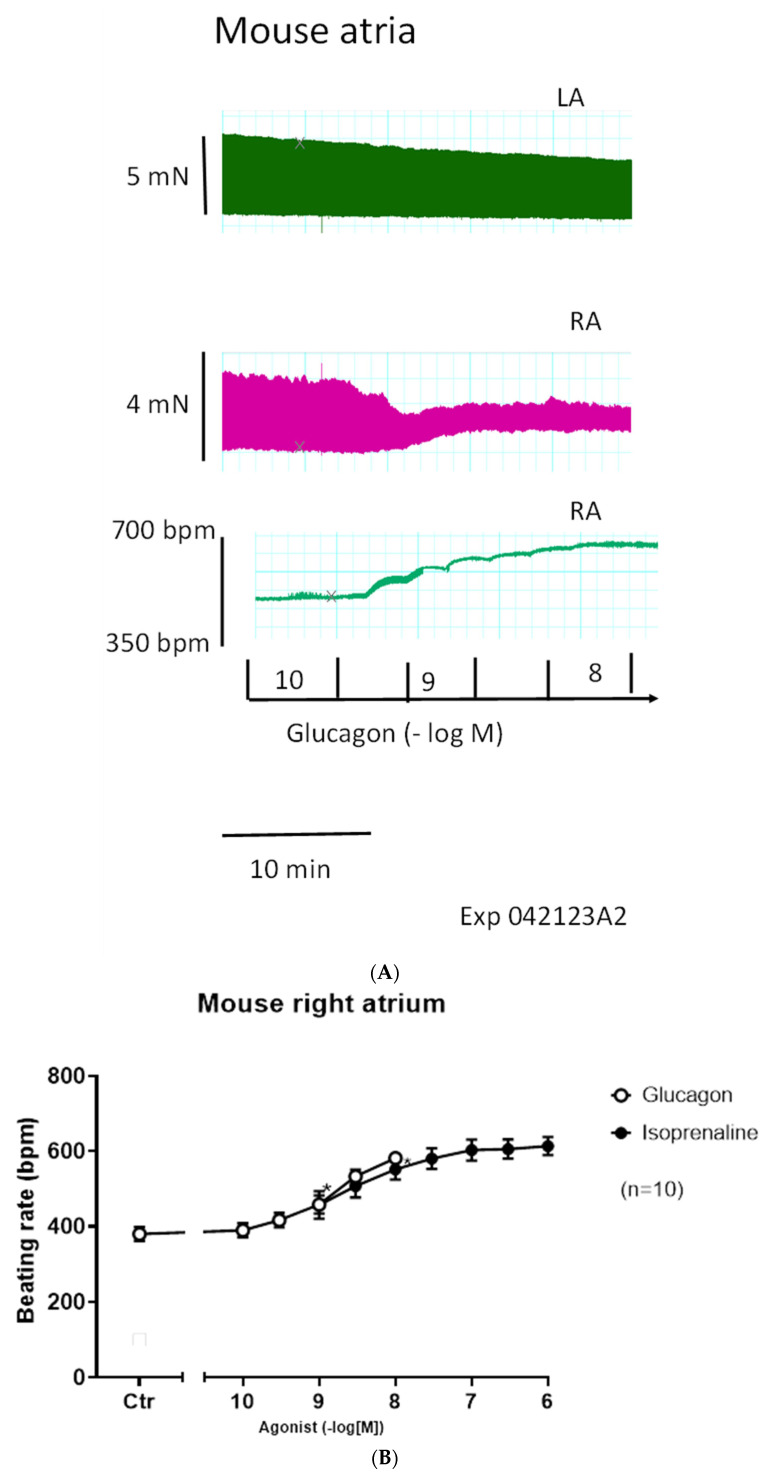

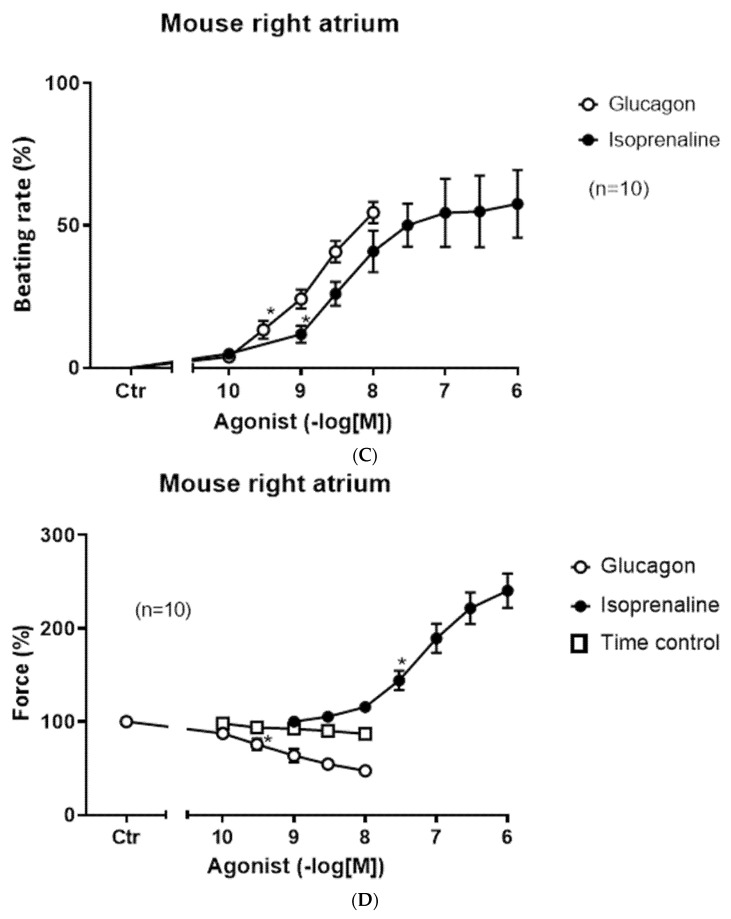

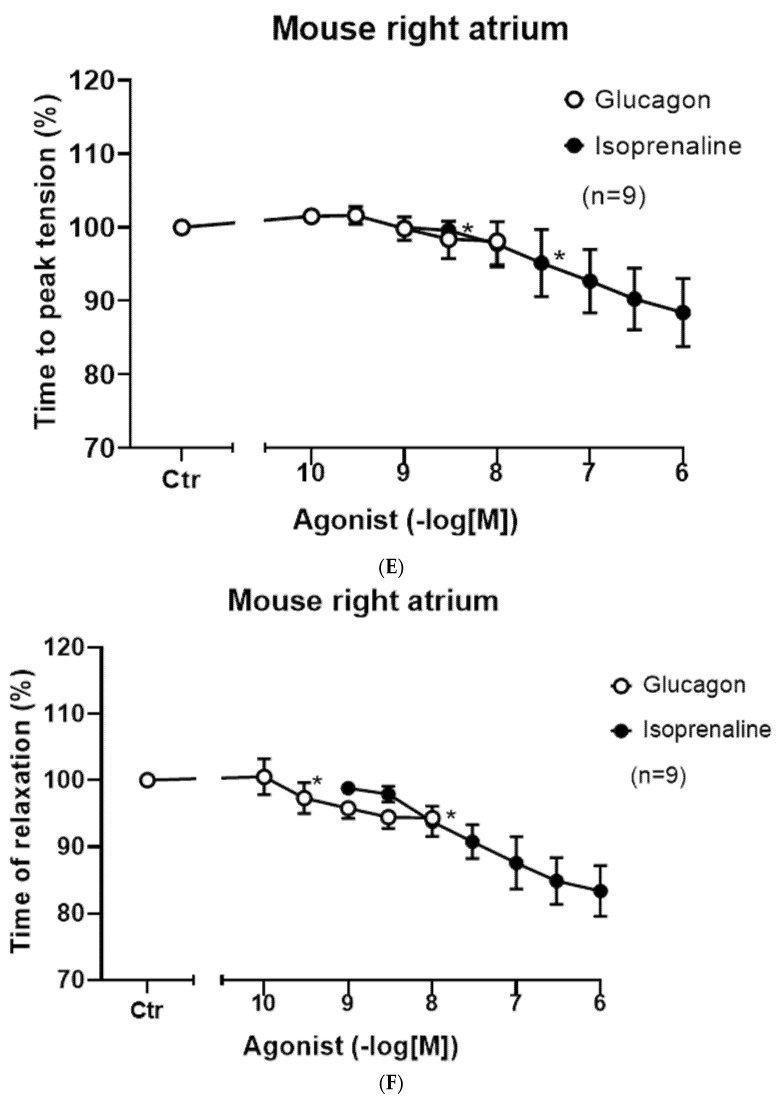
(**A**): Glucagon acts on right but not left mouse atria. (**B**): Glucagon increases the beating rate. Concentration-dependent effects of glucagon (open circles) or isoprenaline (closed circles) on the beating rate in right atrial preparations. Ordinate indicates the beating rate in beats per minute (bpm). * indicates the first significant difference versus the control (Ctr = before drug addition). The number in brackets indicates the number of experiments. Abscissa: concentration of agonists in negative logarithmic molar concentrations. (**C**): Glucagon increases the force of contraction more potently than isoprenaline. Concentration-dependent effect of glucagon (open circles) or isoprenaline (closed circles) as a percentage of the increase in beating rate in right atrial preparations, with reference to pre-drug values. Ordinate indicates the response as a percentage of pre-drug values. Numbers in brackets indicate the number of experiments. Abscissa gives the concentration of agonists in negative logarithmic molar concentrations. Using two-way ANOVA, a *p*-value < 0.05 was calculated between the curves. * indicates the first significant difference versus control (Ctr = before drug addition). (**D**): Glucagon reduces force of contraction. Concentration-dependent effects of glucagon (open circles) or isoprenaline (closed circles) on the force of contraction in right atrial preparations. As a control, we established the force developed over comparable time in right atrial preparations without drug application (time control: squares). Ordinate indicates the force of contraction as a percentage of the pre-drug value (Ctr). * indicates the first significant difference versus Ctr. The number in brackets indicates the number of experiments. Abscissa: concentration of agonists in negative logarithmic molar concentrations. (**E**): Glucagon shortened the time to peak tension. Concentration-dependent effects of glucagon (open circles) or isoprenaline (closed circles) on time to peak tension as a percentage of the pre-drug value (Ctr) in right atrial preparations. Ordinate indicates the time to peak tension as a percentage of Ctr. * indicates the first significant difference versus Ctr. Numbers in brackets = number of experiments. Abscissa: concentration of agonists in negative logarithmic molar concentrations. (**F**): Glucagon shortened the time to relaxation. Concentration-dependent effect of glucagon (open circles) or isoprenaline (closed circles) on time to relaxation in right atrial preparations. Ordinate indicates time of relaxation as a percentage of Ctr. * indicates the first significant difference versus control (Ctr = before drug addition). Abscissa gives the concentration of agonists in negative logarithmic molar concentrations. The number in brackets indicates number of experiments.

We started to compare the effects of glucagon on force of contraction in left atrial preparations and right atrial preparations. One can see in the original tracings of Figure 2A (top tracing) that glucagon failed to increase the force of contraction in mouse left atrial preparations. However, at the same concentrations, glucagon exerted a positive chronotropic effect in right atrial preparations. This positive chronotropic effect of glucagon is seen in original recordings in Figure 2A (lower tracing). In the very same right atrial preparation, where glucagon increased the beating rate, glucagon reduced the force of contraction (Figure 2A, middle tracing).

Original recordings (Figure 2A) of the force of contraction are depicted from electrically stimulated left atrial preparations (top tracing) and the force of contraction in spontaneously beating right atrial preparations (middle). Beating rate (beats per minute, bpm) in the right atrial preparation is displayed in the lower tracing. Glucagon was cumulatively applied. Ordinates give the force of contraction in millinewtons (mN) in the top and middle tracing. The ordinate in the lower tracing displays the beating rate in beats per minute (bpm). The horizonal bar gives the time in minutes (min). We repeated these experiments several times, and mean values are presented in Figure 2B (filled circles).

Isoprenaline exerted a positive chronotropic effect in right atrial preparations (Figure 2B, open circles). Glucagon and isoprenaline were approximately equieffective at raising the beating rate (Figure 2B). We plotted the percentile increase in the beating rate with reference to pre-drug values in Figure 2C. Glucagon was more potent for elevating the beating rate than isoprenaline (Figure 2B). Both glucagon and isoprenaline augmented the beating rate (Figure 2B). Isoprenaline in contrast to glucagon stimulated the force of contraction in right atrial preparations (Figure 2C, closed circles).

In contrast to its effect on the beating rate, glucagon diminished the force of contraction in isolated right atrial preparations (Figure 2D, open circles). As a control, we have plotted (Figure 2D, squares) the force of contraction in right atrial preparations without any drug addition (time control: squares) in time-matched right atrial preparations, which can be compared with the effects of added glucagon (Figure 2D, open circles). Under these conditions where glucagon lessened and isoprenaline raised the force of contraction in right atrial preparations (Figure 2D), glucagon and isoprenaline shortened the time to peak tension (Figure 2E). Glucagon (open circles) and isoprenaline (closed circles) reduced the time of relaxation in right atrial preparations (Figure 2F).

**Figure 3:**
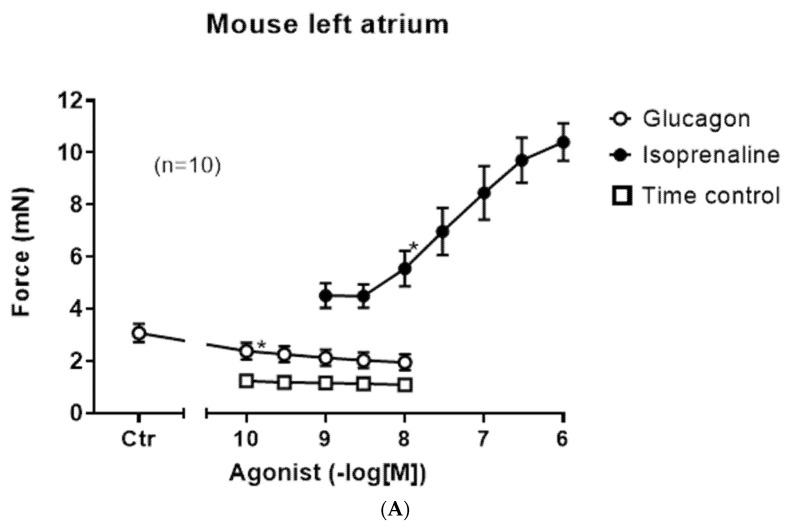

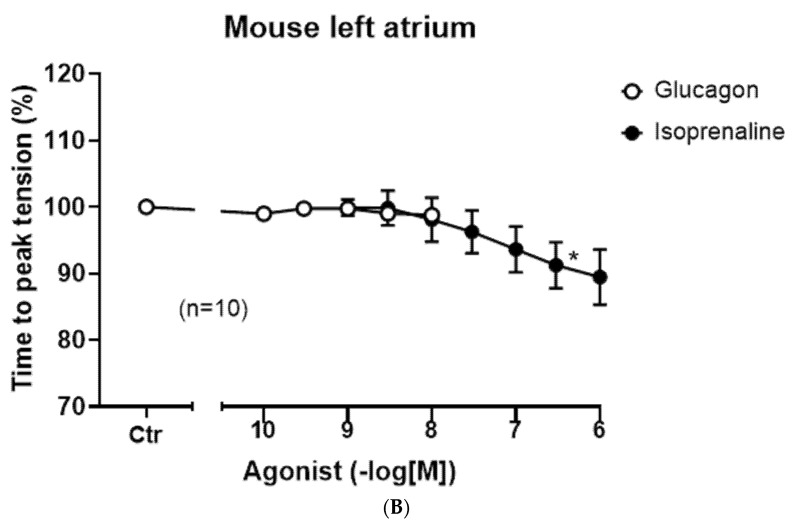
(**A**): Glucagon does not increase the force of contraction in the left atrium. Concentration-dependent effects of glucagon (open circles) or isoprenaline (closed circles) in left atrial preparations. As a control, we established the force developed over comparable times in left atrial preparations without any drug application (time control: squares). Ordinate indicates force in millinewtons (mN). The number in brackets indicates the number of experiments in each group. * indicates the first significant difference versus the control (Ctr = before drug addition). Abscissa gives the concentration of agonists in negative logarithmic molar concentrations. (**B**): Effect of glucagon on the time to peak tension in the left atrium. Concentration-dependent effect of glucagon (open circles) or isoprenaline (closed circles) on the time to peak tension as a percentage of Ctr in left atrial preparations. Ordinate indicates time to peak tension as a percentage of the control (Ctr = before drug addition). * indicates the first significant difference versus Ctr. The number in brackets indicates the number of experiments for each group. Abscissa gives the concentration of agonists in negative logarithmic molar concentrations.

In left atrial preparations, isoprenaline exerts a positive inotropic effect (closed circle), but glucagon did not lead to a positive inotropic effect (Figure 3A, open circles). We also performed control contraction experiments where no glucagon was added to the organ bath at comparable time points (Figure 3A, squares), indicating that the contraction over time is quite stable in the isolated paced mouse left atrium. As expected, isoprenaline in these left atrial preparations shortened the time to peak tension (Figure 3B, closed circle); however, glucagon did not affect the time to peak tension (Figure 3B, open circles).

**Figure 4:**
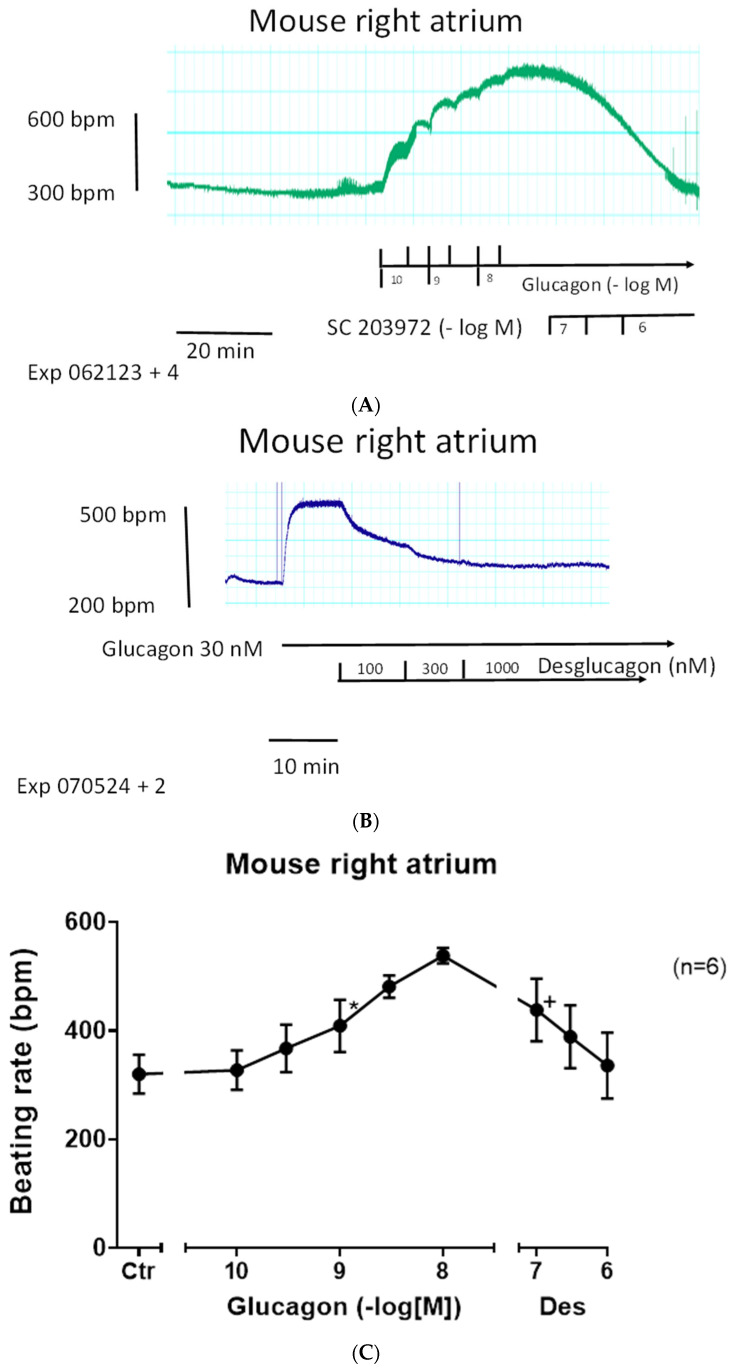

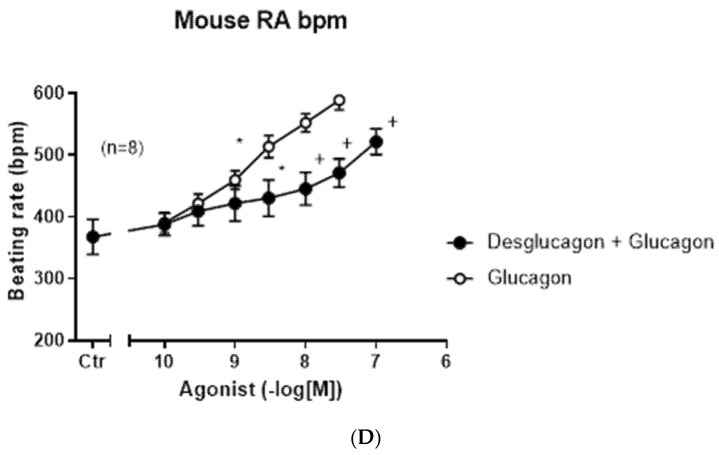
(**A**): Glucagon receptor-antagonist reduces the positive chronotropic effect of glucagon. Original recording of the time-dependent and concentration-dependent positive chronotropic effect of glucagon on the beating rate in mouse right atrial preparations. Where indicated, increasing concentrations of the glucagon receptor-antagonist SC203972 were additionally applied. The horizontal bar indicates time in minutes (min). The vertical bar gives the beating rate in beats per minute (bpm). Short vertical arrows just below the original recording on the left give the time for which glucagon was given in negative logarithmic molar concentration. Lower horizontal arrows on the right give the concentration of SC203972 in negative logarithmic molar concentration. (**B**): GCGR-antagonist reduces the positive chronotropic effect of glucagon. Original recording of the time-dependent positive chronotropic effect of 30 nM of glucagon on the beating rate in mouse right atrial preparations. Where indicated, increasing concentrations of the GCGR-antagonist desglucagon were applied. The lower horizontal bar indicates the time in minutes (min). The vertical bar gives the beating rate in beats per minute (bpm). The top horizontal arrow gives the time for which glucagon was given. The lower horizontal arrow gives concentration of desglucagon in nanomolar (nM) concentrations. (**C**): GCGR-antagonist desglucagon (Des) reduces the positive chronotropic effect of glucagon. Concentration-dependent effects of glucagon (closed circles) on the beating rate in right atrial preparations. * and + indicate a significant difference versus Ctr (Ctr = before drug addition) or versus 10 nM of glucagon, respectively. The number in brackets indicates the number of experiments. Abscissa: concentration of glucagon in negative logarithmic molar concentrations, alone or after additionally applied desglucagon. (**D**): GCGR-antagonist desglucagon reduces the positive chronotropic effect of glucagon. Concentration-dependent effects of glucagon alone (closed circles) or in the additional presence of 100 nM of desglucagon on the beating rate in right atrial preparations. Ordinate indicates the beating rate in beats per minute (bpm). * indicates first significant differences versus Ctr (Ctr = before drug addition), and + indicates first significant differences of glucagon and desglucagon (closed circles) versus corresponding concentrations of glucagon alone (open circles). The number in brackets indicates the number of experiments in each group. Abscissa: concentration of agonist in negative logarithmic molar concentrations of glucagon alone (open circles) or after previously additional application of desglucagon (closed circle).

Next, we asked which receptor is involved in the positive chronotropic effect of glucagon in the right atrium. We used two selective GCGR antagonists, a small organic molecule (SC203972) [25] and the peptide desglucagon [26]. The effect of glucagon to augment the beating rate could be attenuated by subsequent application of the glucagon antagonist SC203972 (Figure 4A). In separate experiments, we first applied 1 µM of SC203972 for 10 min and then added glucagon cumulatively and measured the beating rate: glucagon alone had an EC_50_-value for the increase in the beating rate in right atrial preparations of 8.7 (−lg M) (*n* = 16), which was shifted to the right by SC203972 to an EC_50_-value of 7.8 (−lg M) (*n* = 6, *p* < 0.05).

We could also reduce the positive chronotropic effect of glucagon with a chemically different GCGR-antagonist. After glucagon increased the beating rate, we subsequently applied desglucagon, a peptide antagonist of GCGR. This is displayed in an original tracing in Figure 4B and summarized in Figure 4C.

Moreover, we altered the order of drug addition in further experiments. Here, first 1 µM of desglucagon was applied to the organ bath for 10 min and then increasing concentrations of glucagon were cumulatively added. As depicted in Figure 4D, desglucagon shifted the concentration response curve to the right (Figure 4D, open circles). However, no plateau for the effects of glucagon in the presence of desglucagon was obtained (Figure 4D, closed circles). Hence, no EC_50_-values or pA_2_-values can be reported here. Moreover, one could speculate that once glucagon is produced in the heart or remains in heart (coming from the circulation), then desglucagon might decrease the beating rate. This, however, was not the case: when the basal beating rate under control conditions (no drugs applied) amounted to 350 ± 33 bpm, then an additional 1 µM of desglucagon led to a beating rate of 360 ± 25 bpm after 10 min, which was not significantly different (*n* = 4 each, *p* > 0.05).

**Figure 5:**
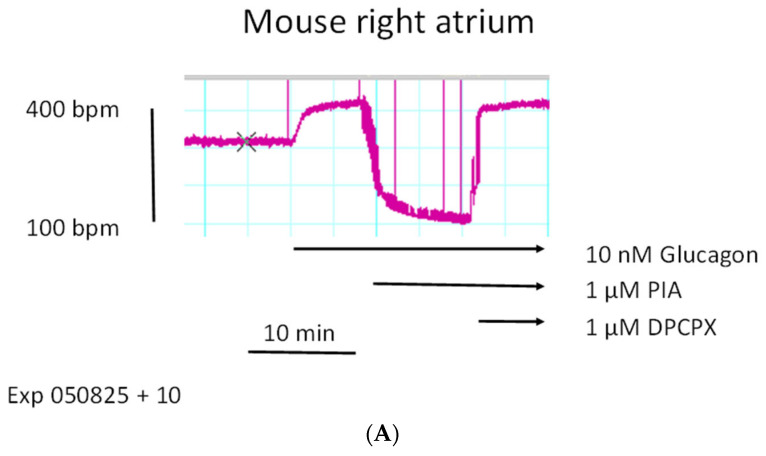

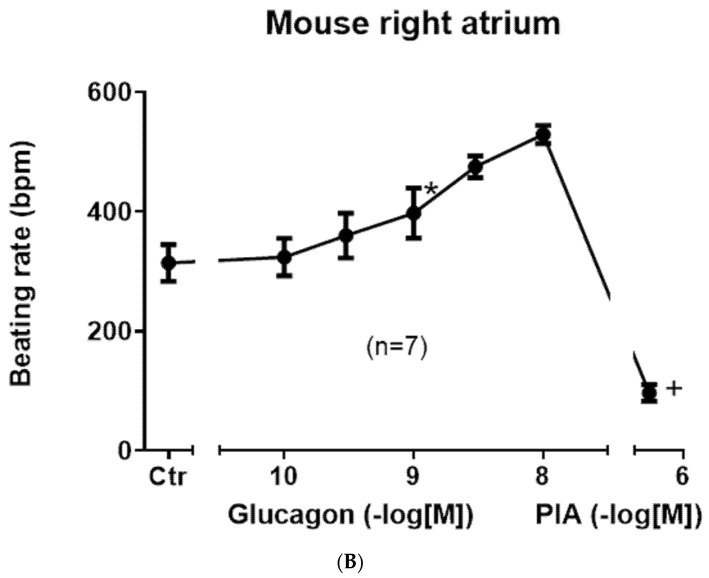
(**A**): R-PIA reduces the positive chronotropic effects of glucagon. Original recording of the time-dependent positive chronotropic effect of glucagon and negative chronotropic effect of R-PIA (PIA) on the beating rate in mouse right atrial preparations. Where indicated by arrows, first PIA and thereafter DPCPX were applied. The vertical bar indicates the beating rate in beats per minute (bpm). Horizontal arrows indicate the time at which the incubation with glucagon, then PIA and finally DPCPX started and was continued thereafter. (**B**): R-PIA reduces the positive chronotropic effects of glucagon. Positive chronotropic effect of glucagon alone and then after addition of R-PIA (PIA). Ordinate indicates the beating rate in beats per minute (bpm). * and + indicate a significant difference versus Ctr (Ctr = before drug addition) or 10 nM of glucagon, respectively. The number in brackets indicate the number of experiments. Abscissa: negative logarithmic molar concentrations of glucagon or PIA.

Next, we report here that the positive chronotropic effects of glucagon in right atrial preparations were antagonized by R-PIA (PIA), an A_1_-adenosine receptor agonist. This is seen in an original recording in Figure 5A. First glucagon raised the beating rate, then additionally applied R-PIA reduced the beating rate. This effect was reversed by DPCPX, an antagonist at A_1_-adenosine receptors (Figure 5A). This is summed up in Figure 5B. We have used R-PIA and DPCPX at these concentrations before in mouse atrial preparations. Next, we noted that carbachol reduced the positive chronotropic effects of glucagon in a right atrial preparation (compare scheme in Figure 1).

**Figure 6:**
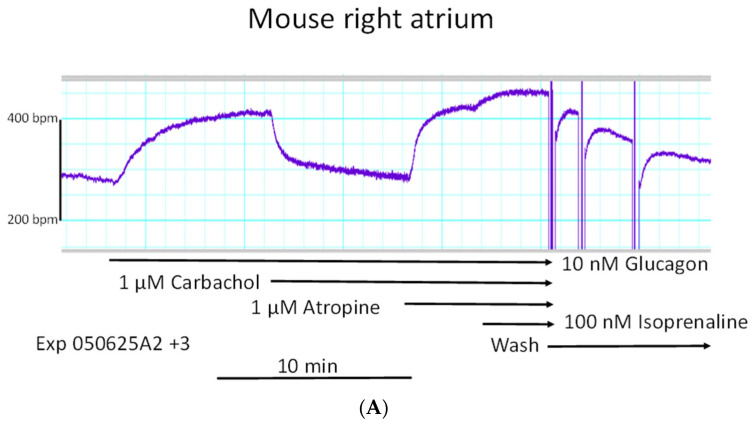

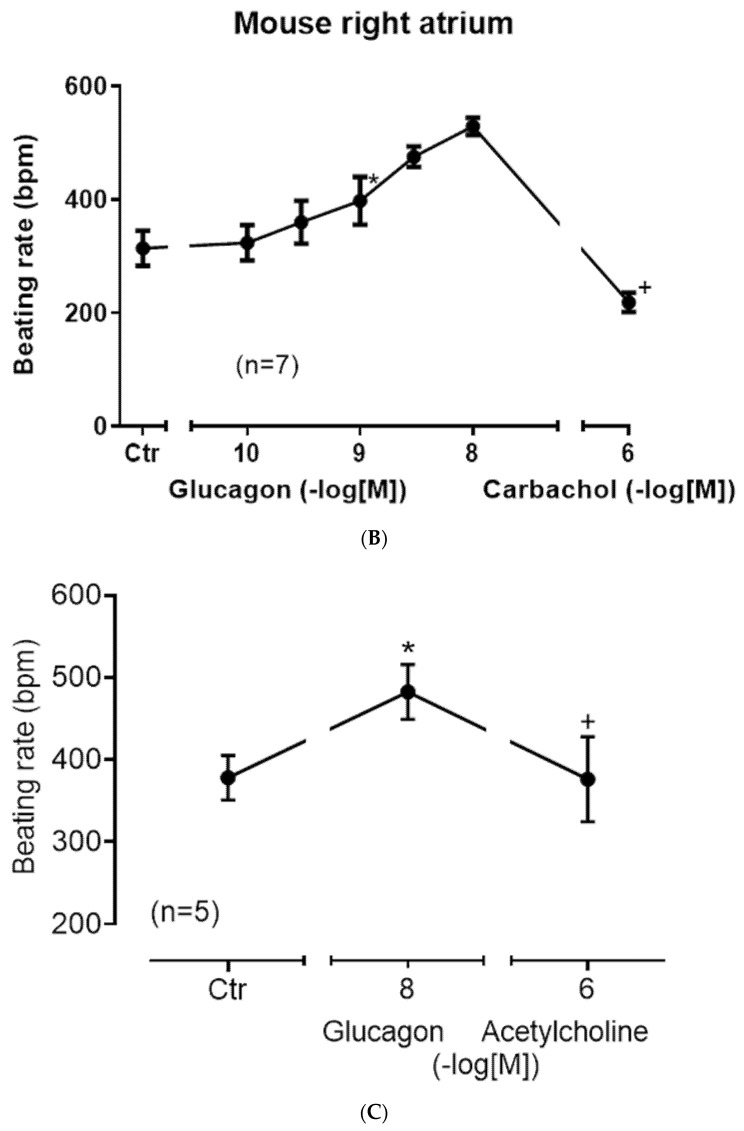
(**A**): Carbachol reduces the positive chronotropic effects of glucagon. Original 10 nM of glucagon (top horizontal arrow) on the beating rate in mouse right atrial preparations. Thereafter, 1 µM of carbachol reversed the glucagon-elevated beating rate (second horizontal arrow). This effect of carbachol was stopped by 1 µM of atropine (third horizontal arrow), and the beating rate climbed again. The beating rate could be raised somewhat further by 100 nM of isoprenaline (fourth horizontal arrow). This elevated beating rate declined after several sequential changes of the buffer in the organ bath with drug-free buffer (wash, bottom horizontal arrow). The horizontal bar indicates the time in minutes (min). The vertical bar gives the beating rate in beats per minute (bpm). (**B**): Carbachol reduces the positive chronotropic effects of glucagon. Concentration-dependent effect of glucagon (closed circles) alone or after addition of 1 µM of carbachol on the beating rate in right atrial preparations. Ordinate indicates the beating rate in beats per minute (bpm). * and + indicate a significant difference versus Ctr (Ctr = before drug addition) or 10 nM of glucagon, respectively. The number in brackets indicates the number of experiments. Abscissa: negative logarithmic molar concentrations of glucagon or carbachol. (**C**): Acetylcholine reduces the positive chronotropic effects of glucagon. Effect of glucagon alone or after addition of acetylcholine on the beating rate in right atrial preparations. * and + indicate a significant difference versus Ctr (Ctr = before drug addition) or 10 nM of glucagon, respectively. The number in brackets indicates the number of experiments. Abscissa: negative logarithmic molar concentrations of glucagon or acetylcholine. Ordinate indicates the beating rate in mouse right atrial preparations in beats per minute (bpm).

This effect is exemplified in an original recording in Figure 6A. The negative chronotropic effect of carbachol was antagonized by the muscarinic receptor antagonist atropine (Figure 6A). This negative chronotropic effect of carbachol after the positive chronotropic effect of carbachol in such experiments is summarized in Figure 6B.

**Figure 7:**
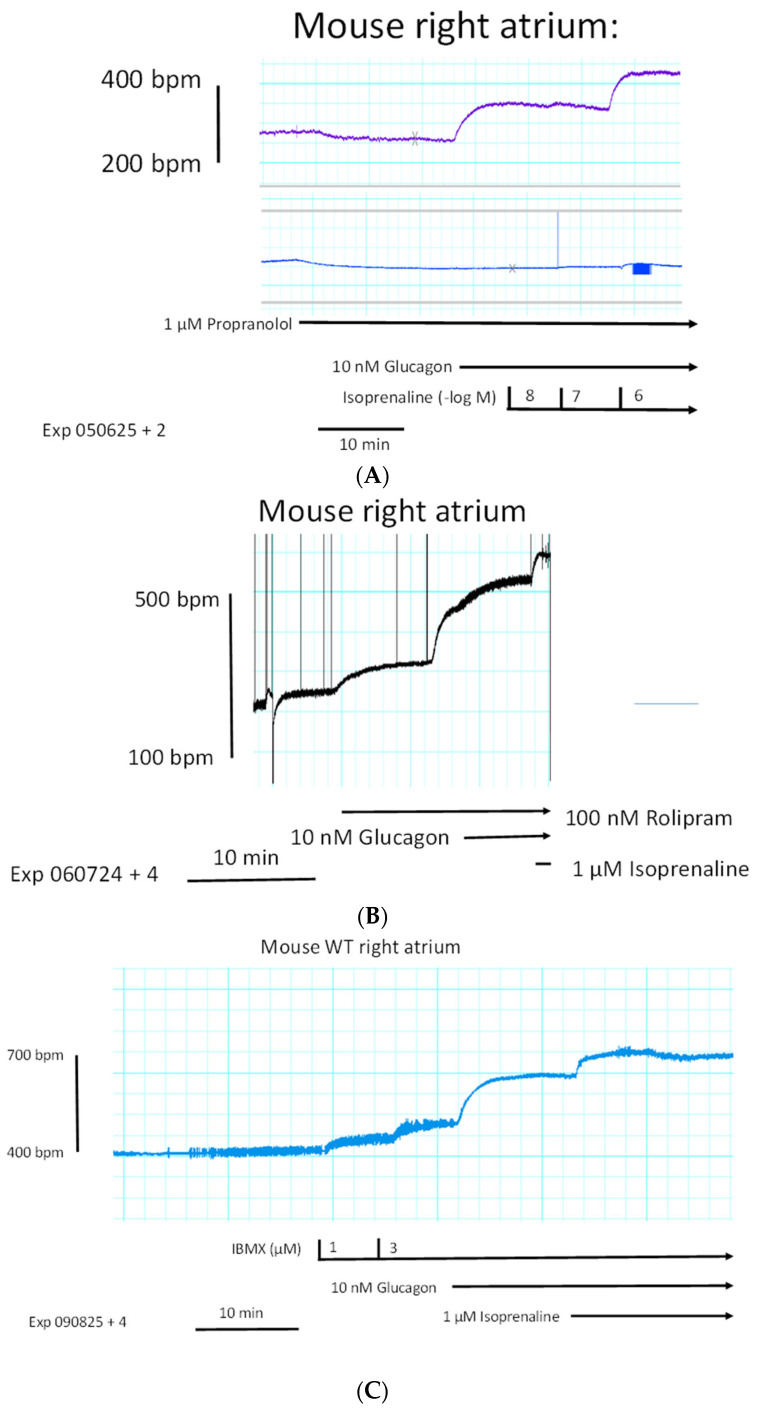

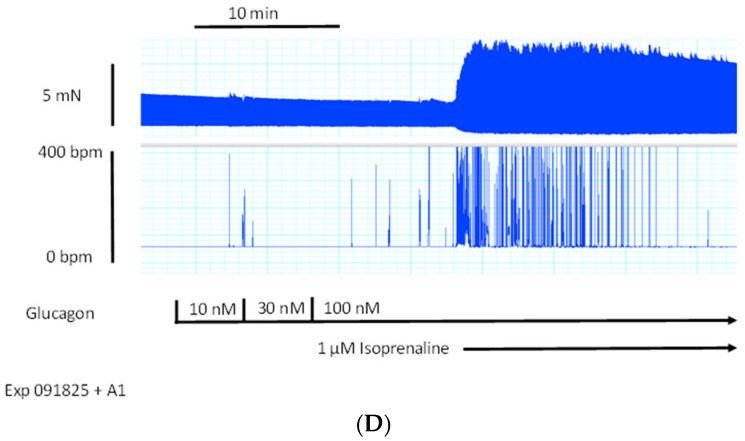
(**A**): Glucagon does not act via β-adrenoceptors. Original recording of the effect of glucagon (second horizontal arrow) in the presence of 1 µM of propranolol (top horizontal arrow) on the beating rate in isolated mouse right atrial preparations. Thereafter, increasing concentrations (in negative logarithmic molar concentrations, small vertical bars) of isoprenaline (bottom horizontal arrow) were added. The left vertical bar gives the beating rate in beats per minute (bpm). (**B**): Glucagon does not act via phosphodiesterase IV. Original recording of the effect of glucagon (second horizontal arrow) in the presence 100 nM of rolipram (top horizontal arrow) on the beating rate in isolated mouse right atrial preparations. Thereafter, isoprenaline was added (right lowest bar). The vertical bar gives the beating rate in beats per minute (bpm). The left horizontal bar gives the time in minutes (min). (**C**): Glucagon does not act via IBMX-sensitive phosphodiesterases. Original recording of the effect of glucagon (middle horizontal arrow) in the presence of increasing concentrations (in µM, short vertical bars) of IBMX (top horizontal arrow) on the beating rate in isolated mouse right atrial preparations. Thereafter, isoprenaline was added (bottom arrow). The left vertical bar gives the beating rate in beats per minute (bpm). The left bottom horizontal bar indicates the time in minutes (min). (**D**): Glucagon does not alter the force of contraction in electrically stimulated right atrial preparations. Some right atrial preparations failed to beat spontaneously. These were paced electrically just like the left atrial preparations. Under these conditions, force was generated (upper ordinate: force of contraction in millinewtons (mN)). Due to the external pacing, the beating rate was constant at the paced 60 beats per minute (lower ordinate in bpm). Then, increasing concentrations of glucagon (in nM, top arrow) were added but failed to alter force (upper tracing) or beating rate (lower tracing). However, when 1 µM of isoprenaline was added (bottom arrow), thereafter force increased (upper tracing) and intermittent arrhythmias were noted (lower tracing). The top horizontal bar indicates the time in minutes (min).

Furthermore, instead of carbachol, we used the endogenous neurotransmitter acetylcholine itself and obtained the same result: acetylcholine diminished the positive chronotropic effect of glucagon as seen in Figure 6C. In order to test whether glucagon might act as an indirect sympathomimetic agent, we studied a possible effect of propranolol (scheme in Figure 1). When we first applied 1 µM of propranolol, a concentration of propranolol which was active because it slightly reduced beating rate (Figure 7A), we noted that 10 nM of glucagon could still increase the beating rate. One might argue that 1 µM of propranolol was too low to completely block β-adrenergic effects of noradrenaline, which might have been released by glucagon from cardiac stores (Figure 1). Hence, we added increasing concentrations of isoprenaline. We noted that 10 nM of isoprenaline, the same concentration we used for glucagon, was ineffective at raising the beating rate. Even 100 nM of isoprenaline did not raise the beating rate (Figure 7A). Only 1 µM of isoprenaline augmented the beating rate (Figure 7A). Hence, release of noradrenaline is unlikely to explain the positive chronotropic effects of glucagon under our experimental conditions. In some species, including mice, glucagon might raise cAMP levels by inhibiting the activity of phosphodiesterases [27,28]. However, when we gave rolipram, a phosphodiesterase 4 inhibitor, additionally applied glucagon could still augment the beating rate (Figure 7B). As a further control, we applied in separate experiments increasing concentrations of the unselective phosphodiesterase inhibitor 3-isobutyl-methylxanthine (IBMX). In addition to phosphodiesterase 4, this drug also inhibits phosphodiesterase 2 and phosphodiesterase 3 [29]. However, while IBMX alone was able to augment the beating rate (Figure 7C), additional glucagon was still able to raise the beating rate further (Figure 7C). This means 10 nM of glucagon was able to enhance the beating rate by 78 ± 12% (*n* = 5, *p* < 0.05) compared to the pre-glucagon values (this is 3 µM of IBMX). This finding is inconsistent with the hypothesis that glucagon acts via inhibition of phosphodiesterases [27]. We have used 100 nM of rolipram and 3 µM of IBMX before in mouse right atrial preparations to raise the beating rate [30]. Others have reported that phosphodiesterase inhibitors can in principle heighten the positive inotropic effect of glucagon in rat ventricular preparation. Hence, cardiac effects of glucagon can be augmented by phosphodiesterase inhibitors, at least in some species [30]. There is evidence from other groups that, in principle, phosphodiesterases can act locally on subcellular compartments of cAMP; this mechanism may come into play under our experimental conditions [31,32]. Moreover, one can ask how glucagon acts, if the right atrium is not beating spontaneously. These right atrial preparations that failed to beat spontaneously were electrically stimulated. Here, the beating rate was set at 60 beats per minute (Figure 7D, lower ordinate). Under these conditions, the electrically stimulated right atrial preparation generated force of contraction (Figure 7D, upper ordinate). When we added increasing concentrations of glucagon that would augment the beating rate in spontaneously beating right atrial preparations (cf. Figure 6B), we did not observe any augmentation in the force of contraction. As a positive control, we next added isoprenaline. Isoprenaline, as expected, raised the force of contraction (upper ordinate), indicating that the mouse right atrium has intact β-adrenoceptors, which, when isoprenaline is added, can elevate force. Please also note that the isoprenaline-induced short-lasting, spontaneous contractions which appear as arrhythmias in the lower ordinate tracing of Figure 7D. In summary, 100 nM of glucagon did not alter the force of contraction in electrically stimulated right atrial preparations of adult mice (amounting 102 ± 9% of pre-glucagon value, *p* > 0.05, *n* = 5).

Next, one can question via which ion channel glucagon augmented the beating rate in right atrial preparations.

**Figure 8:**
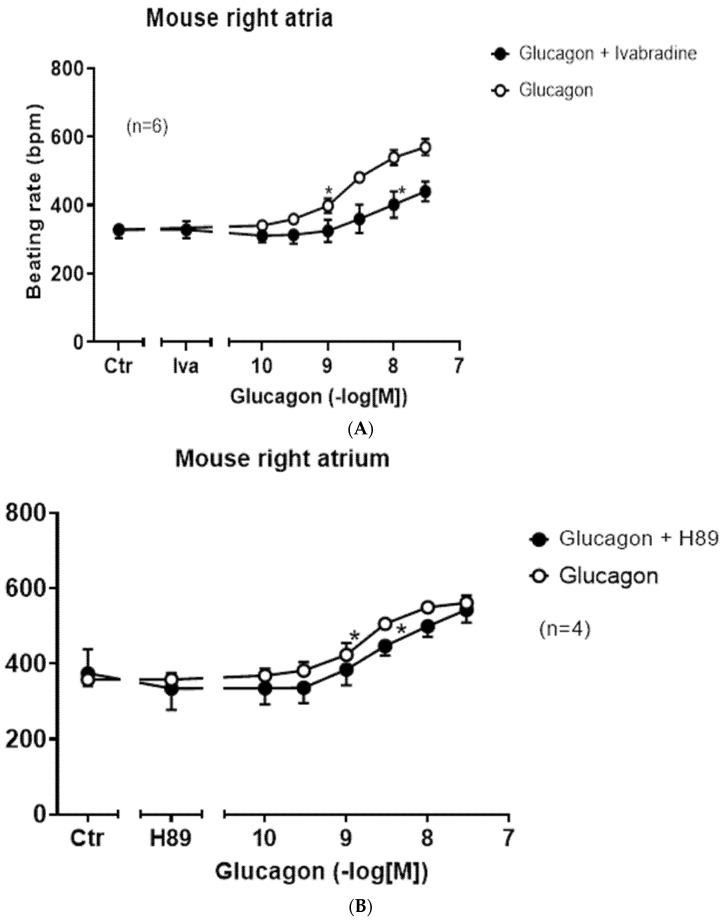

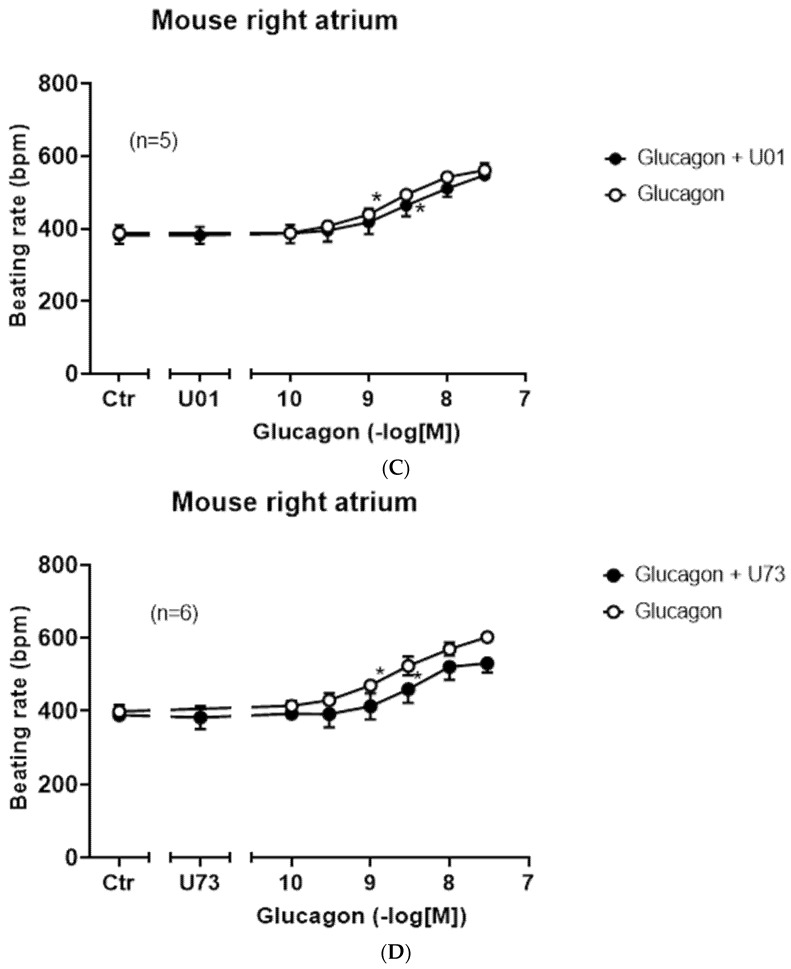
(**A**): Glucagon increases the beating rate via HCN. Concentration-dependent effect of glucagon alone (open circles) or in the presence of 1 µM of ivabradine (Iva, closed circles) on the beating rate in mouse right atrial preparations. Ordinate indicates the beating rate in beats per minute (bpm). * indicates the first significant difference versus Ctr (Ctr = before drug addition). The number in brackets indicates the number of experiments: six in each condition. Abscissa: concentration of glucagon in negative logarithmic molar concentrations of glucagon. Iva means the beating rate after ivabradine. Using two-way ANOVA, a *p*-value < 0.05 was calculated between the curves. (**B**): Glucagon does not act via cAMP-dependent protein kinase. Concentration-dependent effects of glucagon alone (open circles) or in the presence of 10 µM of H89 (closed circles) on the beating rate in mouse right atrial preparations. * indicates the first significant difference versus Ctr (Ctr = before drug addition). The number in brackets means four experiments for each condition. Abscissa: concentration of glucagon in negative logarithmic molar concentrations. H89 indicates the effect of H89 alone. Using two-way ANOVA, a *p*-value of 0.92 was calculated between the curves. (**C**): Glucagon does not act via MAPK. Concentration-dependent effect of glucagon alone (open circle) or in the presence of 10 µM of U0126 (U01, closed red circle) on the beating rate in mouse right atrial preparations. * indicates the first significant difference versus the control (Ctr = before any drug addition). Numbers in brackets indicate the number of experiments in each condition. U01 in the abscissa indicates the effect of 10 µM of U0126 alone. Abscissa: concentration of glucagon in negative logarithmic molar concentrations. Using two-way ANOVA, a *p*-value of 0.62 was calculated between the curves. (**D**): Glucagon does not act via phospholipase C. Concentration-dependent effects of glucagon alone (open circle) or in the presence of 10 µM of U73122 (closed circle) on the beating rate in mouse right atrial preparations. * indicates the first significant difference versus Ctr (Ctr = before drug addition). The number in brackets gives the number of experiments. Abscissa: concentration of glucagon in negative logarithmic molar concentrations of glucagon. U73 in the abscissa indicates the effect of 10 µM of U73122 alone. Using two-way ANOVA, a *p*-value of 0.34 was calculated between the curves.

One likely target, based on the work of others, is the HCN channel, which causes the funny current (Figure 1, I_f_-current, rat: [33]). We chose an HCN channel inhibitor that is an approved drug (against certain forms of chest pain and heart failure), namely ivabradine (1 µM), which we have studied before in mice at this concentration [34]. These results are summed up in Figure 8A. Ivabradine attenuated the effect of glucagon. This can be seen in Figure 8A as a rightward shift of the concentration response curve to glucagon after pre-incubation with 1 µM of ivabradine (Figure 8A). This suggested to us that the positive chronotropic effect of glucagon is mediated, at least in part, by the HCN channels. The cAMP in the right atrium may directly open HCN channels, independently of any phosphorylation of the HCN channels. However, there are reports to the opposite, in which the cAMP-dependent protein kinase may phosphorylate HCN channels [35]. Therefore, we tested the effect of H89, an inhibitor of the cAMP-dependent protein kinase (Figure 8B). Under our experimental conditions, the positive chronotropic effect of glucagon was not attenuated by H89. We have used H89 successfully before to inhibit the activity of the cAMP-dependent protein kinase in the mouse atrium [36]. There are reports that a MAP kinase (MAPK) might mediate biochemical effects of GCGR [37]. Hence, we tested a MAPK inhibitor, which we have used before at this concentration [38], namely 10 µM of U0126 (Figure 1). However, the positive chronotropic effect of glucagon was not affected by pre-incubation for 10 min with 10 µM of U0126, and thus MAPK does not appear to be involved (Figure 8C). Others have reported that glucagon might stimulate the activity of phospholipase C (hepatocytes: [39]). However, 10 µM of U73122, which we used successfully before at this concentration in mouse atria [38], did not attenuate the positive chronotropic effects of glucagon in isolated right atrial preparations from adult wild-type hearts (Figure 8D).

**Figure 9:**
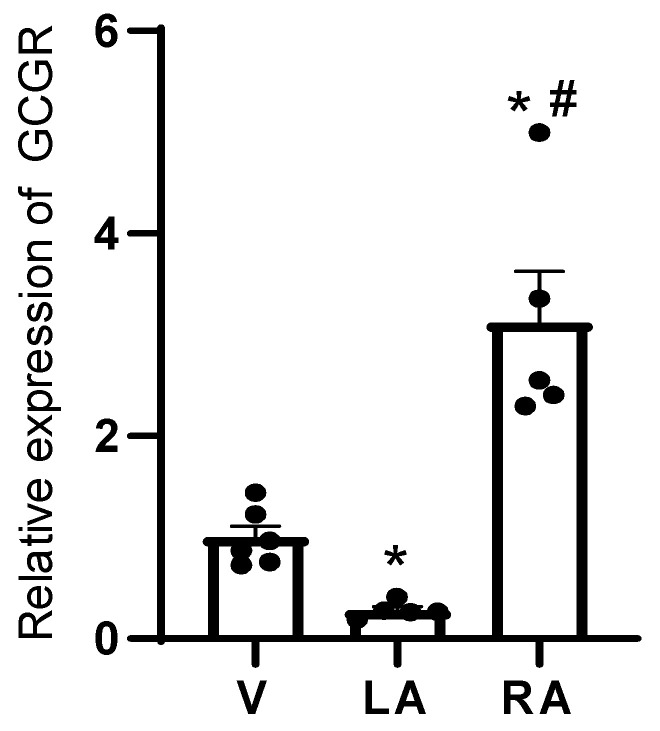
High gene expression of endogenous mouse glucagon receptor (GCGR) in the right atrium. The amount of the mRNA for GCGR in the right atrium (RA), in the left atrium (LA) and in the ventricle (V) of adult murine hearts. Data are presented as arithmetic means ± SEM with scatter plots of individual data points, *n* = 5–6 per group; * *p* < 0.05 vs. V, # *p* < 0.05 vs. LA. Ordinate indicates gene expression of the GCGR in various regions of the mouse heart relative to a house-keeping gene (peptidylprolyl isomerase A).

Moreover, the question arose of whether the positive chronotropic effects of glucagon were only seen in isolated right atrial preparations or were a phenomenon also noticeable in more clinically relevant, more physiological preparations. Hence, we infused glucagon retrogradely via the coronary arteries into the isolated spontaneously beating mouse heart (Langendorff heart: Table 1). The main result was that, as in the isolated right atrium, in the whole mouse heart, glucagon also augmented the beating rate (Table 1). Here, we performed a post hoc power analysis. We calculated the power to detect an augmentation in the beating rate of 98%. Interestingly, the contractile data in the left atrium agree with the contractile data in the mouse right atrium: in the same isolated mouse hearts where glucagon raised the beating rate, glucagon also reduced the force of contraction (Table 1). This can be explained by the negative staircase (“Treppe”) phenomenon: a rise in beating rate per se attenuated the force of contraction in the left ventricle of the adult mouse heart under our experimental conditions. We also noted in the whole perfused heart that glucagon reduced the time to peak tension and time to relaxation (Table 1). Finally, we studied the gene expression of the GCGR in the regions of the mouse heart. The expression was highest in the right atrium, hardly detectable in the left atrium and small in the mouse ventricle (Figure 9).

Here, we also performed a post hoc power analysis. We calculated a power of 99% to detect an increase in the amount of mRNA for the GCGR between LA and RA.

## 3. Discussion

The main new finding of this report is that glucagon exerts a positive chronotropic effect via GCGR in adult mouse right atrial preparations. One can ask why glucagon raised the beating rate but not the force of contraction in spontaneously beating right atrial preparations of adult mice, whereas isoprenaline could augment the beating rate and force under these conditions. It is conceivable that the GCGR is only present in the sinus node, whereas the β-adrenoceptors are present in the sinus node and in the remainder of the right atrium. This view is supported by our studies in paced right atria (Figure 7D). Another major finding was that also in left atrial preparations, we failed to find a positive inotropic effect of glucagon, whereas under the same experimental conditions, isoprenaline elevated the force of contraction. These findings are difficult to reconcile with the mechanism plotted in Figure 1. Why should glucagon augment cAMP only in the right atrium and not in the left atrium? A rise in cAMP in the right atrium explains any augmentation in activity of the HCN and thus the climbing of the beating rate in the right atrium (Figure 1). A rise in cAMP in the left atrium should activate the regulatory proteins responsible for force generation in the left atrium. However, this finding is not without precedence: prostaglandin E1 raised cAMP in kitten hearts and beating rate but not force of contraction, which might have been caused by subcellular compartments of cAMP [40].

It merits some thoughts why glucagon failed to elevate the force of contraction in adult mouse left atrial preparations. The action of glucagon in the left atrium probably does not lead to an augmentation of cAMP-levels in contrast to the sinus node but may use a different signal transduction pathway. Alternatively, the GCGR might not be expressed in the cardiomyocytes of the mouse left atrium but in other left atrial cells, for instance in endothelial cells. Consistently, we measured only small amounts of GCGR in the left atrium, which might indicate a very low or absent expression of GCGR in cardiomyocytes (Figure 9). But this GCGR might be located on endothelial cells, and thus it cannot raise cAMP in left atrial cardiomyocytes. This would explain why glucagon does not augment the force of contraction in adult mouse left atrial preparations. Interestingly, not only in the mouse left atrium (this paper) but also in the rat left atrium, glucagon failed to raise the force of contraction [33]. Hence, this lack of left atrial inotropy to glucagon may be a general phenomenon in rodents. As concerns the mechanism underlying the positive chronotropic effect of glucagon, we would like to mention that we had studied ivabradine before in mouse right atrial preparations and noted a negative chronotropic effect [34]. In extension, here ivabradine attenuated the positive chronotropic effect of glucagon. From our present data, we assume that glucagon acts in the sinus node of adult mice via stimulation of HCN channels. This stimulation of HCN channels then would elevate the heartbeat. This glucagon-stimulated heartbeat was reversed by carbachol and R-PIA. In other species than mice, glucagon exerts a positive inotropic effect in cardiac preparations. For instance, the positive inotropic effects of glucagon in the isolated canine ventricle were antagonized by carbachol or acetylcholine; however, the effects of glucagon on the spontaneous beating rate of the canine right atrium were not reported [41,42]. As another example, in rat ventricular cells, acetylcholine could reduce the glucagon-stimulated current through the L-type calcium ion channel [28]. Hence, a cardiac antagonism between glucagon and acetylcholine is not without precedence in the mammalian heart. Here, a further new finding is that such an antagonism is also present in the mammalian sinus node. The observation that carbachol reduced the positive chronotropic effect of glucagon in mouse right atrial preparations suggests that this interaction is similar to that in rat ventricular cardiomyocytes or canine ventricle. Hence, we extend previous findings to a new species (mouse) and a new tissue (atrium) and other contractile parameters (beating rate). It is now well known that acetylcholine can be produced in the heart [43]. Thus, in a paracrine way, cardiac acetylcholine might reduce conceivably detrimental glucagon-induced tachycardia in mice. As far as we know, it is a further novel finding that an A_1_-adenosine receptor agonist like R-PIA can reduce any glucagon-induced effect in mammalian sinus nodes. However, this is biochemically not without precedence in other tissue: R-PIA inhibited glucagon-stimulated activity of adenylyl cyclase in rat Sertoli cells and in hepatic cells [44,45,46]. Hence, in principle, an interaction of the A_1_-adenosine receptor and the GCGR seems to exist and is now extended to the mouse sinus node. Like acetylcholine, adenosine is also produced in the heart [47]. This production of cardiac adenosine rises in ischemia and reperfusion. Hence, our findings (Figure 5B) open the possibility that adenosine like acetylcholine offers a protection against the putative detrimental tachycardia caused by glucagon, especially in cardiac ischemia. As concerns the signal transduction in the sinus node of mice, our data would argue for an involvement of cAMP at the HCN channel but do not support an action via the cAMP-dependent protein kinase because H89 was without effect (Figure 9). Likewise, the positive chronotropic effects of glucagon in adult mice are probably not mediated via MAPK (Figure 9). Likewise, we failed to detect evidence for an involvement of phospholipase C in the positive chronotropic effects of glucagon (Figure 9).

Of note, we show here that glucagon not only acted in the isolated mouse right atrium. Importantly, glucagon also elevated the beating rate in isolated perfused mouse hearts (Table 1). This might be interpreted as being consistent with a positive chronotropic effect of injected glucagon in living mice. This is mechanistically interesting, as our data would argue that there is also a direct cardiac effect of glucagon in living mice. However, there is GCGR in the brain, and our data do not rule out that this brain GCGR also contributes to a positive chronotropic effect of injected glucagon in living mice. One has to admit that the concentrations of glucagon we used are elevated and thus here represent pharmacological concentrations; i.e., these concentrations are much higher than the physiological concentrations of the glucagon that are found in humans or experimental animals (around 10 pM [15]). However, like us, many other groups used nanomolar or even micromolar concentrations of glucagon to elicit a positive chronotropic or positive inotropic effect in other species [13,33,48].

We have recently reported that glucagon alone had no positive inotropic effect in human atrial preparations. However, in the presence of a phosphodiesterase inhibitor 3 inhibitor, namely cilostamide, glucagon augmented the force of contraction in human atrial preparations [49]. Actually, this finding was one reason why we studied phosphodiesterase inhibitors here. We had shown that only in the presence of rolipram (a phosphodiesterase 4 inhibitor; phosphodiesterase 4 is the main phosphodiesterase in the mouse heart, and phosphodiesterase 3 is the main phosphodiesterase in the human heart) did some drugs exert positive inotropic effects. This is probably the case because phosphodiesterase inhibitors induced a rise in cAMP in such a way that even small further increases in cAMP by receptor stimulation can add up to a positive inotropic effect. This seems to be the case in the human atrium. Another issue is that human right atrial preparations, which we routinely study, very rarely beat spontaneously. In such rare cases, we noted in human right atrial preparations from patients suffering from persistent atrial fibrillation that their isolated right atrial preparations (which do not include the human sinus node) initially beat spontaneously. However, usually after three times of changing the buffer in the organ bath, which is our standard procedure, the isolated human right atrial preparations stopped beating on their own, and we had to start to pace them. In short, we could not study sinus node function in our isolated human right atrial preparations. Hence, we studied as a surrogate parameter the sinus node function in the right atrium of the mouse heart.

Furthermore, the present data give a plausible reason why we failed to detect a positive inotropic effect in the left atrium of the mouse heart: the mRNA of the glucagon receptor is very low in the mouse left atrium. In contrast, others have published that the mRNA of the glucagon receptor is present in human right atrial samples. Indeed, as just mentioned, we measured a positive inotropic effect of glucagon in the presence of cilostamide in human right atrial preparations. Hence, in humans, the functionally active GCGR is apparently present in high levels on cardiomyocytes in the human right atrium (and not endothelial cells). Thus, glucagon can elevate the force in isolated human right atrial muscle preparations even outside the sinus node [50]. Hence, we would argue that the inotropic effect in the human atrium of glucagon can be explained by high regional GCGR expression in cardiomyocytes. The most straightforward explanation for the lack of a positive inotropic effect of glucagon in the mouse left atrium is the very low expression of the glucagon receptor, and this expression might be confined to endothelial cells. The situation might be different in the mouse right atrium: we detect high levels of mRNA for glucagon receptor in the right atrium (Figure 9). We note a positive chronotropic effect of glucagon in the mouse right atrium but interestingly no positive inotropic effect in the paced mouse right atrium. This may mean that the GCGR is only expressed in the sinus node in the mouse right atrium and not in the remaining muscle part of the right atrium (which is different qualitatively and quantitatively from the human expression, as discussed above).

**Clinical relevance**: It is probable that pharmacological concentrations of glucagon can induce a direct positive chronotropic effect in humans. This effect is probably GCGR-mediated. Thus, there might be a clinical perspective to our present studies in mice. We have shown that glucagon augmented the force of contraction in the isolated electrically human right atrial muscle strips via GCGR, and hence such a pathway exist, in principle [49]. Thus, glucagon might act directly on the human heart via GCGR to augment force and beating rate. However, this is still controversial and merits further work, e.g., with human-pluripotent-stem-cell-derived cardiomyocytes, whether glucagon can directly elevate the heartbeat in the human heart.

Limitations of the study: We did not have the opportunity to measure the expression of the GCGR in the sinus node of mice and compare this expression with the expression of the GCGR in the remainder of the right atrium of the adult mouse. We have not been able to measure the effects of glucagon on cAMP microdomains in the mouse right atrial cardiomyocytes or sinus node cardiomyocytes. We have not yet measured whether glucagon raised the I_f_-current in the sinus node of adult mice. Hence, due to our technical boundaries, we cannot prove with certainty the signal transduction of the GCGR in the mouse sinus node. Moreover, we have not removed the sinus node in control experiments on isolated perfused hearts and then measured pressure in the left ventricular with external pacing to understand the role of glucagon in the ventricle more definitely. In addition, one might isolate ventricular cardiomyocytes from adult wild-type mice, pace them electrically and then study the effect of glucagon on contractility. As other control experiments for future studies, it would be useful to pre-treat living mice with reserpine or 6-hydroxy-dopamine to empty cardiac noradrenaline stores. Finally, we have not yet performed comparison contraction experiments on right atrial preparations from adult mice with a cardiac or general GCGR deletion. If our assumption of a GCGR-mediated effect of glucagon is valid, in these mice lacking GCGR, glucagon should not lead to a positive chronotropic effect.

In conclusion,

1. Glucagon increased the beating rate in the mouse heart.
2. This was mediated by cardiac GCGR acting probably at least in part through stimulation of HCN channels.

## 4. Materials and Methods

### 4.1. Contractile Studies in Mice

Breeding, keeping and handling of mice complied with local legal requirements (permit number I8M9). In brief, adult wild-type mice (CD1), bred at our institution, of both sexes (21 male mice and 22 female mice with a mean age of 10.2 months and a mean weight of 40.2 ± 8.8 g) were sacrificed by cervical dislocation; the thorax was cut open, and the heart was moved forward and meticulously dissected from the aorta in order not to damage the right atrium. It took about ten minutes from cervical dislocation to the start of the contractions in the organ bath. We have four to eight organ baths at our disposal. We use these baths to load from one mouse the left atrium in the first organ bath and the right atrium into the second organ bath, from the second mouse the left atrium in the third organ bath and so on. Moreover, we normally used littermate wild-type mice on each experimental day to reduce systematic errors. The experimenter was not blinded to the drug that was added, because glucagon induced a potent positive chronotropic effect and no inotropic effect. On each experimental day, all organ baths were filled with Tyrode’s solution, which was gassed in a separated thermostat of 2000 mL volume, so that the buffer in each organ bath should be identical. We define the right or left auricula as a right or left atrial preparation. The bathing solution of the organ baths, a modified Tyrode’s solution, contained (in mM) 119.8 NaCI, 5.4 KCI, CaCl_2_, MgCl_2_, 0.42 NaH_2_PO_4_, 22.6 NaHCO_3_, 0.05 Na_2_EDTA, 0.28 ascorbic acid and 5.05 mM glucose. Ascorbic acid was included in the buffer to protect, e.g., isoprenaline against oxidation (e.g., [21]). A gas containing 95% O_2_ and 5% CO_2_ streamed continuously through the buffer in the organ to keep the pH at 7.4 [21]. The right or left atrial preparations, cut as delineated above, were fixed with metal clips on a rod within the organ baths [38]. In isolated left atrial preparation, the force of contraction was measured routinely under electrical stimulation (using platinum electrodes). Here, we stimulated with biphasic rectangular impulses for 5 ms. The voltage usually amounted to five volts, which was about 10% higher than required to start muscle contraction at one beat per second (1 Hz). Spontaneously beating right atrial preparations in mice were used to study any chronotropic effects. Some right atrial preparations did not beat spontaneously but had to be electrically stimulated exactly as left atrial preparations (Figure 7C). Force was measured under isometric conditions, amplified using bridge amplifiers (AD Instruments, Oxford, UK) and measured by means of commercial software (Lab Chart 8, AD Instruments, Oxford, UK). This software was used to calculate force in millinewtons (mN), the time to peak tension and the time to relaxation. To the double-barrelled organ baths (10 mL volume), we added glucagon in parallel, cumulatively or non-cumulatively.

### 4.2. Langendorff Perfusion

As described by our group [36], isolated whole mouse hearts were retrogradely perfused with a modified Tyrode solution comprising (in mM) 119.8 NaCI, 5.4 KCI, CaCl_2_, MgCl_2_, 0.42 NaH_2_PO_4_, 22.6 NaHCO_3_, 0.05 Na_2_EDTA, 0.28 ascorbic acid and 5.05 mM glucose in a non-recirculating manner. The modified Tyrode solution was warmed and gassed before the perfusion. The perfusion was performed in a non-recirculating mode with a peristaltic pump such that the flow into the heart was two millilitres in a minute. Hearts were beating spontaneously. Force was monitored at the apex cordis using a fixed hook. The hook was connected with a thin thread to a force detector, digitized and analysed (Labchart 8 from AD Instruments). This was used to calculate the first derivative of force with respect to time and to assess the maximum rate of relaxation and the maximum rate of force development. Glucagon was kept in a syringe which was connected to the inlet of the pumping system. This syringe was used to apply the drug to the aorta.

### 4.3. Polymerase Chain Reaction (PCR)

We pulverized frozen samples of murine left and right atrial preparations and murine ventricles in liquid nitrogen. Afterwards, we extracted RNA using the acid phenol–guanidinium thiocyanate–chloroform procedure (TRI Reagent^®^, Sigma-Aldrich, St. Louis, MO, USA) as described by the manufacturer. We precipitated the RNA from the aqueous phase by application of isopropanol in a 1:1 ratio to the extracted RNA. After centrifugation (11,000 rpm, 4 °C, 30 min), we obtained RNA pellets. We washed these pellets twice with ethanol. We tested the integrity of the extracted RNA. To this end, the RNA was subjected to electrophoresis using two percent agarose-containing gels (agarose, Sigma-Aldrich, USA). Intact RNA samples underwent reverse transcription with the help of a high-capacity cDNA reverse transcription kit with RNAse inhibitors (Applied Biosystems, Grand Island, NY, USA). We performed a quantitative real-time PCR (RT-qPCR) analysis using the QuantStudio™ 3 Real-Time PCR System (Thermo Fisher Scientific, Waltham, MA, USA) with SYBR™ Select Master Mix (Thermo Fisher Scientific, USA). We assessed the expression of the glucagon receptor (Gcgr gene) in mouse ventricles and left and right atria. We used forward and reverse primers for the Gcgr gene as follows: the forward primer TGGTACCACAAAGTGCAGCA and the reverse primer TCCAACTGACATTGGGAGGC. The Pfaffl method [51] was used to calculate the relative expression. The results were normalized to the reference gene Ppia (peptidylprolyl isomerase A). Here, we used a forward primer with the sequence GCGTCTCCTTCGAGCTGTTT and a reverse primer with the sequence CACCCTGGCACATGAATCCT. We designed the primers with the help of Primer-BLAST (http://www.ncbi.nlm.nih.gov/tools/primer-blast, accessed on 4 December 2025) [52]. Calculated normalized quantities were calibrated to appropriate V group (ventricles).

### 4.4. Data Analysis

We show arithmetic means ± standard error of the mean to facilitate comparison with our previous contractile studies, for instance of retatrutide [53,54,55]. We assessed the statistical significance of the difference between means with the help of analysis of variance followed by Bonferroni’s *t*-test, paired Student’s *t*-tests or two-way ANOVA as we thought appropriate and as described in the figure legends. We regard a *p*-value of less than 0.05 as significant. Experimental data were plotted by choosing sigmoidal curve fitting with the help of the commercial software GraphPad Prism 8. This software was also used when we confirmed that our data were normally distributed. To perform a power analysis, we assumed an alpha error of 5% and used freely available software called G-power (version 3.1.9.7) (www.gpower.hhu.de).

### 4.5. Drugs and Materials

(−)-Isoprenaline (1-(3,4-dihydroxyphenyl)–2-isopropylaminoethanol) (+)-bitartrate, SC203972 (*n*-(3-cyano–6-(1,1-dimethylpropyl)–4,5,6,7-tetrahydro–1-benzothien–2-yl)–2-ethylbutanamide, SQ22536 (9-(tetrahydro–2-furanyl)–9H-purin–6-amine) and ivabradine ((S)–3-{3-[([3,4-dimethoxybicyclo [4.2.0]octa–1,3,5-trien–7-yl]methyl)methylamino]-propyl}–1,3,4,5-tetrahydro–7,8-dimethoxy–2H–3-benzazepin–2-on) were purchased from Sigma-Aldrich (now Merck, Dreieich, Germany), while adomeglivant (LY2409021 3-[[4-[(1S)–1-[4-(4-tert-butylphenyl)–3,5-dimethylphenoxy]–4,4,4-trifluorobutyl]-benzoyl]amino]-propanoic acid, biomol), 8-cyclopentyl–1,3-dipropylxanthine (DPCPX) and DesHis1-Glu9-glucagon 1–29 amide were purchased from Biotechne/Tocris (Wiesbaden, Germany). Human glucagon, (−)N^6^-phenylpropyladenosine (PIA), carbachol and atropine were purchased from Merck (Dreieich, Germany). For the remaining chemicals, we bought only those that were of the highest purity that were commercially available. We employed deionized water to prepare the buffers in our experiments. We prepared the stock solutions of the drugs used freshly on each experimental day.

## Figures and Tables

**Table 1:** Glucagon increases the beating rate in isolated retrogradely perfused (Langendorff mode) mouse hearts. Effect of nM of glucagon (after 10 min perfusion) on the force of contraction in millinewtons (mN) and the rate of tension relaxation mN/seconds (mN/s), the rate of tension development (mN/s), time to peak force in milliseconds (ms), time to relaxation (ms) or spontaneous beating rate in beats per minute (bpm) in isolated perfused hearts from adult wild-type mice. Data are given in arithmetic mean values ± standard error of the mean. * indicates glucagon effect *p* < 0.05 versus the appropriate basal value in a paired *t*-test. *n* gives the number of experiments.

*n*	5
Basal force (mN)	17.0 ± 1.7
Force after glucagon (mN)	11.3 ± 1.8 *
Basal rate of tension development (mN/s)	498 ± 59.1
Rate of relaxation after glucagon (mN/s)	410 ± 70.5 *
Basal rate of relaxation (mN/s)	−358 ± 50.3
Rate of relaxation after glucagon (mN/s)	−270 ± 41.6 *
Basal beating rate (bpm)	270 ± 9.5
Beating rate after glucagon (bpm)	446 ± 15.0 *
Basal time to peak force (ms)	34.6 ± 0.9
Time to peak force (ms) after glucagon	29.6 ± 0.7
Basal time of relaxation (ms)	50.0 ± 2.3 *
Time of relaxation (ms) after glucagon	40.6 ± 2.1 *

## Data Availability

The original contributions presented in this study are included in the article. Further inquiries can be directed to the corresponding author.
